# Assessing the Clinical and Socioeconomic Burden of Respiratory Syncytial Virus in Children Aged Under 5 Years in Primary Care: Protocol for a Prospective Cohort Study in England and Report on the Adaptations of the Study to the COVID-19 Pandemic

**DOI:** 10.2196/38026

**Published:** 2022-08-25

**Authors:** Uy Hoang, Elizabeth Button, Miguel Armstrong, Cecilia Okusi, Joanna Ellis, Maria Zambon, Sneha Anand, Gayathri Delanerolle, F D Richard Hobbs, Jojanneke van Summeren, John Paget, Simon de Lusignan

**Affiliations:** 1 Nuffield Department of Primary Care Health Sciences University of Oxford Oxford United Kingdom; 2 Imperial College London London United Kingdom; 3 Reference Microbiology Services United Kingdom Health Security Agency London United Kingdom; 4 Netherlands Institute for Health Services Research Utrecht Netherlands

**Keywords:** medical records systems, computerized, respiratory syncytial virus, general practitioners, pandemics, COVID-19, general practice, primary health care, outcome assessment, health care, respiratory, children, pediatric

## Abstract

**Background:**

Respiratory syncytial virus (RSV) commonly causes lower respiratory tract infections and hospitalization in children. In 2019-2020, the Europe-wide RSV ComNet standardized study protocol was developed to measure the clinical and socioeconomic disease burden of RSV infections among children aged <5 years in primary care. RSV has a recognized seasonality in England.

**Objective:**

We aimed to describe (1) the adaptations of the RSV ComNet standardized study protocol for England and (2) the challenges of conducting the study during the COVID-19 pandemic.

**Methods:**

This study was conducted by the Oxford-Royal College of General Practitioners Research and Surveillance Centre—the English national primary care sentinel network. We invited all (N=248) general practices within the network that undertook virology sampling to participate in the study by recruiting eligible patients (registered population: n=3,056,583). Children aged <5 years with the following case definition of RSV infection were included in the study: those consulting a health care practitioner in primary care with symptoms meeting the World Health Organization’s definition of acute respiratory illness or influenza-like illness who have laboratory-confirmed RSV infection. The parents/guardians of these cases were asked to complete 2 previously validated questionnaires (14 and 30 days postsampling). A sample size of at least 100 RSV-positive cases is required to estimate the percentage of children that consult in primary care who need hospitalization. Assuming a swab positivity rate of 20% in children aged <5 years, we estimated that 500 swabs are required. We adapted our method for the pandemic by extending sampling planned for winter 2020-2021 to a rolling data collection, allowing verbal consent and introducing home swabbing because of increased web-based consultations during the COVID-19 pandemic.

**Results:**

The preliminary results of the data collection between International Organization for Standardization (ISO) weeks 1-41 in 2021 are described. There was no RSV detected in the winter of 2020-2021 through the study. The first positive RSV swab collected through the sentinel network in England was collected in ISO week 17 and then every week since ISO week 25. In total, 16 (N=248, 6.5%) of the virology-sampling practices volunteered to participate; these were high-sampling practices collecting the majority of eligible swabs across the sentinel network—200 (43.8%) out of 457 swabs, of which 54 (N=200, 27%) were positive for RSV.

**Conclusions:**

Measures to control the COVID-19 pandemic meant there was no circulating RSV last winter; however, RSV has circulated out of season, as detected by the sentinel network. The sentinel network practices have collected 40% (200/500) of the required samples, and 27% (54/200) were RSV positive. We have demonstrated the feasibility of implementing a European-standardized RSV disease burden study protocol in England during a pandemic, and we now need to recruit to this adapted protocol.

**International Registered Report Identifier (IRRID):**

DERR1-10.2196/38026

## Introduction

“Burden of disease” refers to the human and economic costs that result from poor health [1,2]. Respiratory syncytial virus (RSV) “burden of disease” studies in young children (aged <5 years) have mostly been focused on the morbidity and mortality rates of RSV infections [3]. In the United States, most of the health care use related to RSV in children occurs in outpatient settings [4]. Common presentations of RSV including bronchiolitis, bronchitis, pneumonia, and other lower respiratory tract infections (LRTI) are managed in primary care [5]. In the United Kingdom, this pediatric primary care is provided by general practitioners (GPs) [6]. A global burden of disease study estimates that there are 33.1 million young children infected with RSV, resulting in 3.2 million hospitalizations and 59,600 in-hospital deaths [3]; although in western countries, mortality due to RSV is rare and tends to occur in those with underlying risk factors [7].

RSV epidemics occur annually in temperate climates during the winter months, and less consistent epidemics occur in the (sub)tropics [8]. Most studies have found a positive correlation with latitude, as peak RSV activity generally occurs later in the year with increased latitude in both the northern and southern hemispheres [8-10]. One region where this is not the case is Europe, where 3 different studies have found contradictory results [11].

A study from Spain measured health care use related to RSV infections in young children in primary care and calculated the associated costs [12]. A recent literature review found only 2 further studies in primary care that have investigated the clinical and socioeconomic burden of laboratory-confirmed RSV infections in young children [1].

Further information on the clinical and socioeconomic burden of RSV is needed to support the development of clinical services and preventative care for children in the United Kingdom, including the implementation of effective preventative measures against RSV that could reduce the impact of severe LRTI for children and reduce the clinical workload in primary care [13,14].

During the winter of 2019-2020, the “RSV ComNet” team, managed by the Netherlands Institute for Health Services Research, developed a standardized study protocol and patient questionnaires to measure the clinical and socioeconomic disease burden of laboratory-confirmed RSV infections among young children (aged <5 years) in primary care. They initially tested this protocol and validated the questionnaires in Italy and the Netherlands, among 293 and 152 children, respectively, in each country, of which 119 (41%) and 32 (21%) tested positive for RSV, respectively, and 116 and 12 were included for follow-up questionnaires, respectively [1].

This paper describes our adaptations of the “RSV ComNet” standardized study protocol and its validated study questionnaires for use in England—the RSV ComNet II study. We also describe the modifications made to implement the study during the COVID-19 pandemic and evaluate the revised data collection procedures.

The RSV ComNet II study aims to describe the epidemiology of RSV in primary care in England, including the RSV incidence rates and the clinical and socioeconomic disease burden of RSV in children aged <5 years. The objective of this paper was to describe the adaptations to the RSV ComNet standardized study protocol to execute the study in England. In addition, our secondary objectives were to present preliminary results from the RSV season in 2020-2021 and the demographic and clinical characteristics of the study population included so far.

## Methods

The methods are described in 4 parts: (1) the case definition of eligible participants, planned measurements, timing of follow-up questionnaires, and number of participants required; (2) adaptations of the RSV ComNet standardized study protocol in England; (3) adaptations of our approach for the COVID-19 pandemic; and (4) statistical methods and sample size calculation.

### Case Definition of Eligible Patients

We used the following case definition of RSV:

- Children aged <5 years
- Consulting a GP with symptoms meeting the World Health Organization and European Centre for Disease Control’s definitions of acute respiratory illness (ARI) or influenza-like illness (ILI) [15,16], see Table 1
- A reference laboratory–confirmed polymerase chain reaction diagnosis of RSV (antigen testing of swabs is not undertaken)

The following exclusion criteria were applied:

- Parents with insufficient knowledge of English
- Parents who are, for whatever reason, unable to provide informed consent
- Special personal circumstances in the family (based on the judgement of the GP; eg, a recent death in the family) and the lack of informed consent

**Table 1:** ARI^a^ and ILI^b^ case definitions used.

	ARI [17,18]	ILI [16,19,20]
Symptoms	Acute—defined as a sudden onset of symptoms Respiratory infection—defined as having at least one of the following: shortness of breath, cough, sore throat, and coryza Clinician’s judgement that the illness is due to an infection and that there is not a more plausible diagnosis	An acute respiratory illness with a temperature measured, reported, or plausibly ≥38 °C and a cough, with onset within the past 10 days ILI cases have a sudden onset, and symptoms are often suggestive of systemic upset—myalgia, fatigue, malaise, and headache, etc ILI cases should not have another more plausible diagnosis
SNOMED^c^ codes used to track symptoms	Acute bronchitis (SCTID^d^: 10509002) or acute bronchiolitis (SCTID: 5505005), according to whether the infection was judged to be in the upper or lower respiratory tract, respectively	ILI (finding; SCTID: 95891005)

^a^ARI: acute respiratory illness.

^b^ILI: influenza-like illness.

^c^SNOMED: Systematized Nomenclature of Medicine.

^d^SCTID: SNOMED Clinical Terms identifier.

### Planned Measurements and Timing

**Figure 1:**
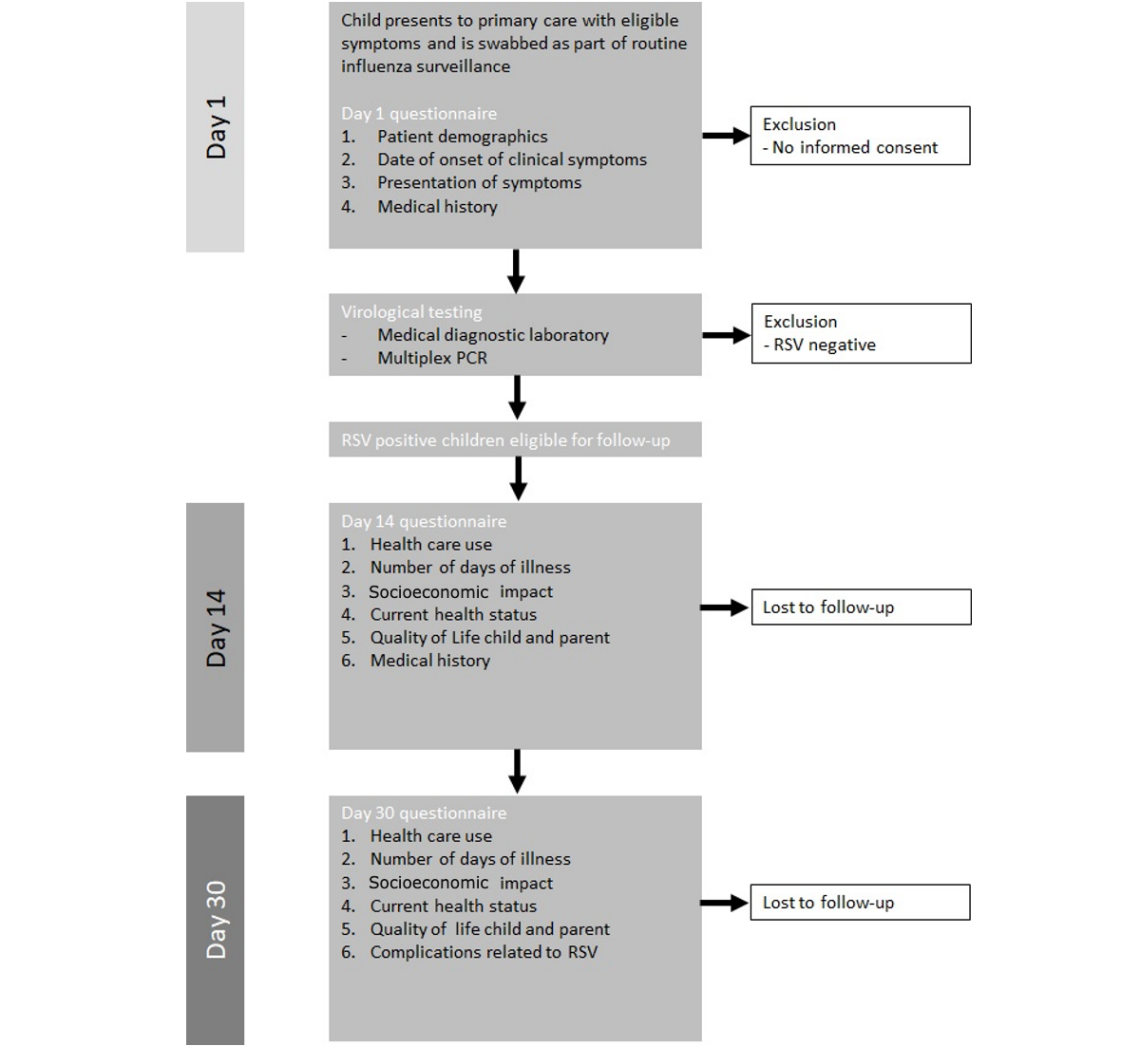
Study schedule of events for RSV ComNet II study in England. PCR: polymerase chain reaction; RSV: respiratory syncytial virus.

We used questionnaires previously evaluated as part of the RSV ComNet study [1]. These questionnaires record the clinical and socioeconomic impact of RSV at 14 and 30 days postswab. Figure 1 shows the RSV ComNet II study schedule of events in England. A copy of the combined Day 14 questionnaire is provided in Multimedia Appendix 1.

Primary care staff conducted the questionnaire follow-ups with the parents/guardians of children aged <5 years with RSV-positive swabs over the telephone, to increase the response rate, rather than sending paper questionnaires to the participants’ home. Responses to the questionnaire were entered electronically by study staff through a dedicated website and stored in a secure database. Questionnaire information from this database will be linked to the computerized medical records (CMR) to analyze the final study results.

At the day of the swab (Day 1), information related to the demographics of the patient, date of symptom onset, presentation of symptoms, past medical history, and viral testing performed were extracted from the CMR and virology swabbing specimen forms of the consenting patients.

At 14 days postswab, questions relating to the health care use of the child within the past 2 weeks, number of days of illness, hospitalizations and accident and emergency department visits, current health status, quality of life, and socioeconomic impact on parents or caregivers were asked.

At 30 days postswab, the parents were asked to complete a final questionnaire similar to the Day 14 questionnaire, with an additional question regarding any complications related to the RSV infection, such as pneumonia or otitis media acute (ear infections) visits within the past month.

### Sample Size Calculation

To estimate the clinical and socioeconomic disease burden of RSV with sufficient precision, it is necessary to have a sufficient sample of RSV-positive patients with a range of disease severity. To identify the optimal feasible sample size for the outcome “hospitalization rate,” the RSV ComNet study team calculated the precision for this outcome, characterized by the 95% CI width, for 3 scenarios [21]. Scenarios were calculated for a sample size of 100, 150, and 200 RSV-positive cases and an expected RSV hospitalization rate of 6%. The corresponding 95% CIs were calculated to be from 1.3% to 10.7% (n=100), from 2.2% to 9.8% (n=150), and from 2.7% to 9.3% (n=200), and therefore, the study team decided that 100 RSV-positive cases were the minimum required feasible sample size.

Assuming a swab positivity rate of 20% in children aged <5 years, we estimated that a total of at least 500 swabs in the children aged <5 years category was required to reach the recommended sample size of 100 RSV-positive patients.

### Specific Methods for Implementing the RSV ComNet II Standardized Study Protocol in England

#### English National Sentinel Surveillance Network

In England, the RSV ComNet II study was embedded within the English national sentinel surveillance network run by the Oxford-Royal College of General Practitioners (RCGP) Research and Surveillance Centre (RSC) [22]. Information from this network has been used to monitor respiratory infections including influenza and RSV for over 50 years [23]. Over this period, practices have had feedback about their data quality around influenza and respiratory disease.

The network consists of >1800 general practices in England, of which 248 take part in virology sampling—using swabs to monitor the spread of respiratory illnesses including COVID-19, influenza, and RSV. Since the COVID-19 pandemic started, virology sampling has taken place all year round.

#### Practice Recruitment for the RSV ComNet II Study in England

We invited all 248 virology-sampling practices within the sentinel surveillance network to participate in the RSV ComNet II study. Those that agree to participate in the study were given training on identifying and collecting consent from eligible patients, adding relevant study codes to the patient’s CMR, and undertaking patient follow-up study questionnaires. Additional guidance was provided for swabbing children aged <5 years if requested.

#### Participant Recruitment for the RSV ComNet II Study in England

The opportunistic recruitment of participants took place in study practices. The parents/guardians of children presenting to their GP with symptoms meeting the study inclusion criteria were approached for consent by their GP or a trained study nurse. If written consent was obtained, then practices were asked to keep a copy of the signed consent form in the practice and record study consent directly into the CMR. Following consent, a nose and throat, or 2 nasal swabs, was taken and sent to the UK Health Security Agency reference virology laboratory for multiplex reverse transcription–polymerase chain reaction testing.

Study practices were encouraged to increase swabbing when RSV was observed to be circulating among sentinel network practices.

#### Oxford-RCGP Clinical Informatics Digital Hub

All participating practices that are part of the Oxford-RCGP RSC sentinel surveillance network have consented to the routine data extraction of information from the CMR into the Oxford-RCGP Clinical Informatics Digital Hub—a trusted research environment [24,25]. For virology specimens, information is collected by specific sentinel network request forms (with an electronic option), and the results are transmitted back to patient CMR through the eLab system (Emulation S.Hein).

Data about participant demographic characteristics and the clinical disease burden of RSV infection will be gathered from the Oxford-RCGP Clinical Informatics Digital Hub, including information from the virology specimens and patient questionnaires.

### Adaptations to the RSV ComNet II Study in England Due to the COVID-19 Pandemic

The study was planned for winter 2020-2021 starting from January 4, 2021 (International Organization for Standardization [ISO] week 1), but the seasonality of RSV was interrupted by the use of nonpharmaceutical interventions (NPIs) such as lockdowns, school closures, social distancing, and the obligatory use of face masks during the winter of 2020-2021 as a result of the COVID-19 pandemic [26,27].

As a result of the NPIs and fewer patients coming to practices for face-to-face consultations, the Oxford-RCGP RSC also set up a parallel system to enable patients to order self-test kits that are sent to their home as part of virology surveillance, which have been shown to be reliable when compared to clinician-led sampling [28-31]. This system was incorporated into the study.

Through the sentinel network, we were able to identify which practices saw many symptomatic children, saw recent RSV-positive cases, and were regularly swabbing in the children aged <5 years category. We identified a positive correlation between the presentations of respiratory symptoms in children versus the number of swabs taken by practices.

We adjusted our practice recruitment strategy to actively target the practices with high RSV swab positivity rates. These practices were approached directly by research facilitators to inform them about the RSV positivity at their practice and invited to participate in the study.

A further adaptation to the study recruitment was to allow for initial verbal consent into the study if the patient was not seen in person.

Specific adaptations to the Day 14 questionnaire were made to facilitate data collection in England. First, all questions from the Day 1 consultation (ie, related to patient demographics, date of onset of clinical symptoms, and presenting clinical symptoms) are included in the Day 14 questionnaire. This inclusion was to ensure that all questions were asked if it was not possible during a time-limited initial consultation or information was missing from the virological swabbing specimen form. The only exception is a question on malnutrition, which the original protocol states should only be collected from the medical record. Second, additional questions on complications related to RSV infection were included in the Day 30 questionnaire, essentially creating a single combined questionnaire for situations where a Day 30 questionnaire was not possible, such as lost to follow-up cases due to pressure on parents/guardians to care for their children.

A further adaptation was made to expand the time window for completing the questionnaires to increase the response rate. If practices struggled to complete them within 14 and 30 days after the swab was taken, an allowance was given to retrospectively contact patients up to 60 days after the swab was taken using the combined Day 14 questionnaire.

Lastly, the study was originally due to end in June 2021. However, due to RSV-positive cases first appearing in April and June 2021, a decision was made to extend recruitment through to September 2021. The study was then extended to cover the winter season from October 2021 to the end of May 2022.

### Statistical Methods

Our analysis describes the deviant RSV season in 2020-2021 including symptom incidence rates in the network, symptom incidence rates in the RSV study practices, swabbing rates in study practices, swab positivity rate in all the virology-swabbing practices within the network and RSV study practices in particular, bronchitis and ILI incidence rates in the network, and survey questionnaire response rates. The descriptive analysis using data collected to date are presented in this paper.

We also investigated differences in symptom incidence rates, swabbing rates, swab positivity rate, RSV incidence rates, and survey questionnaire response rates between the RSV ComNet study practices in England and RSV rates measured in other European countries that implemented the RSV ComNet study protocol. Data are presented graphically by ISO weeks [32].

### Ethics Approval

The study was approved by the English National Research Ethics Committees (Integrated Research Application System: 285025; Research Ethics Committees: 20/PR/0704). Subsequent study adaptions due to the COVID-19 pandemic described above were also granted approval by the English National Research Ethics Committees.

## Results

### Reported Results and Future Analyses

We present results on swabbing rates and swab positivity rates from the study practices and preliminary results from the questionnaires. The results include the demographic and clinical characteristics of young children with RSV infections in primary care. Additional analyses about the clinical and socioeconomic disease burden of RSV infections, including information obtained from the linkage of study questionnaires to the patients’ electronic medical records, and the final analysis of the study are expected to be completed by June 2023.

### Weekly Incidence Rates of ARI and ILI

**Figure 2:**
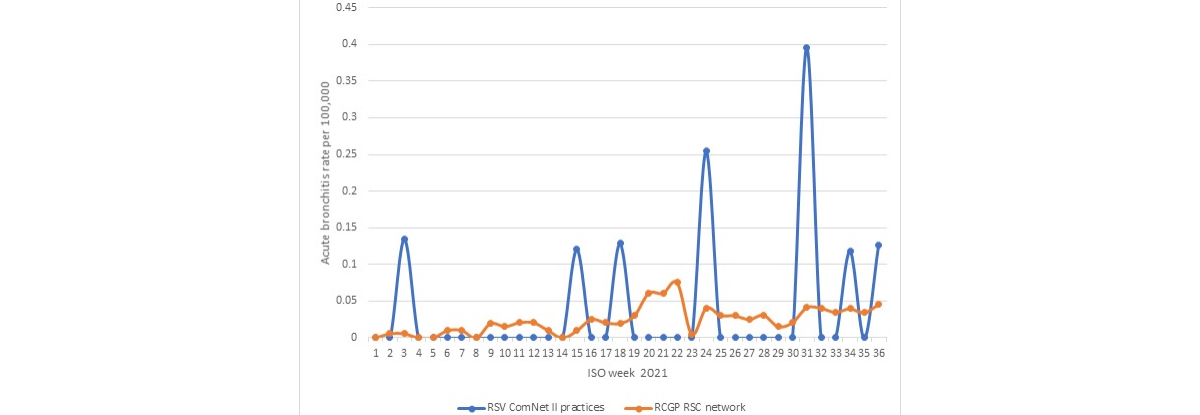
Acute bronchitis (SCTID: 10509002) incidence rate in RCGP RSC network compared with RSV ComNet II study practices. RCGP RSC: Royal College of General Practitioners Research and Surveillance Centre; RSV: respiratory syncytial virus; SCTID: Systematized Nomenclature of Medicine Clinical Terms identifier.

**Figure 3:**
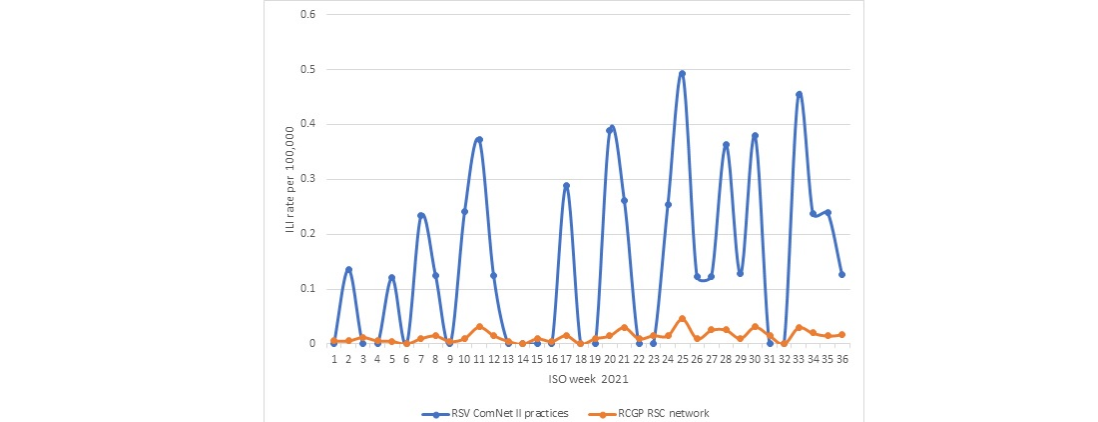
ILI (SCTID: 95891005) incidence rate in RCGP RSC network compared with RSV ComNet II study practices. ILI: influenza-like illness; RCGP RSC: Royal College of General Practitioners Research and Surveillance Centre; RSV: respiratory syncytial virus; SCTID: Systematized Nomenclature of Medicine Clinical Terms identifier.

There was no clear seasonal incidence of acute bronchitis or ILI in the 2020-2021 season. Figures 2 and 3 showed that the incidence rates of both ARI, as denoted by Systematized Nomenclature of Medicine Clinical Terms identifier (SCTID) 10509002—acute bronchitis, and ILI (SCTID: 95891005) across the network fluctuated during the course of the study, which fluctuated much more among RSV ComNet II study practices.

### RSV ComNet II Study Practice Recruitment in England

**Figure 4:**
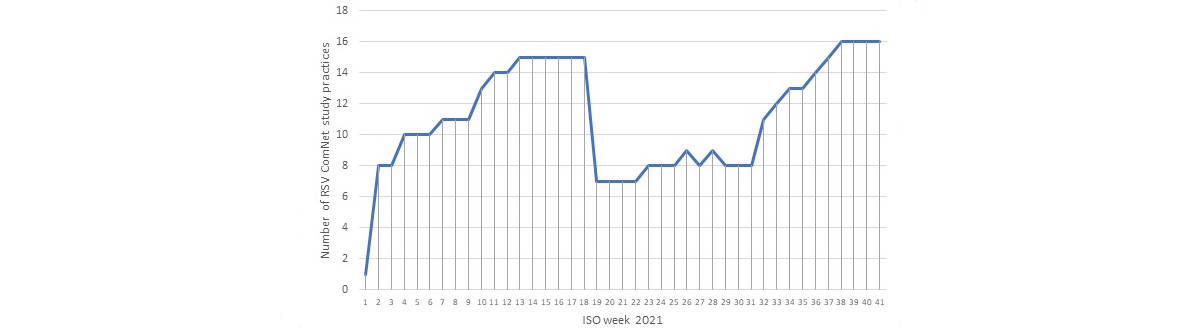
Number of practices recruited to the RSV ComNet II by week. ISO: International Organization for Standardization; RSV: respiratory syncytial virus.

**Figure 5:**
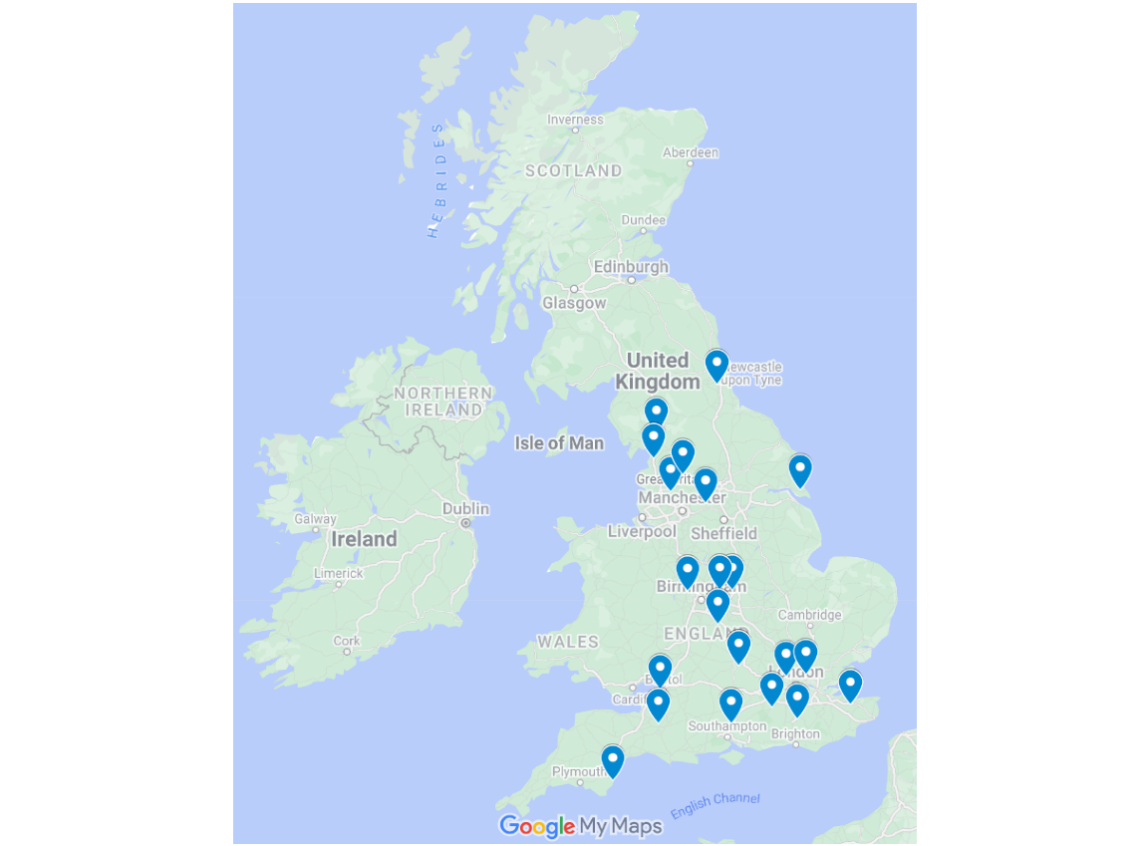
Map of study practice locations.

We recruited 16 practices into the study, with a registered population of 250,333 patients as of ISO week 41, 2021, which equates to 6.5% (16/248) of all virology-sampling practices within the RSC sentinel surveillance network. Figure 4 shows the recruitment of practices to the RSV ComNet II study by week. Figure 5 shows a map of the study practice locations. Between ISO weeks 18 and 19, there was a drop in the number of participating practices, which was due to on-going capacity issues resulting from the pandemic.

### Virology-Sampling Rates and RSV Positivity

In total, 457 swabs in children aged <5 years were collected across all 248 virology-sampling practices in the sentinel surveillance network since January 4, 2021, up to ISO week 41, 2021, of which 100 swabs had been collected across the sentinel surveillance network in the winter season of 2020-2021 between ISO weeks 1-20, 2021.

**Figure 6:**
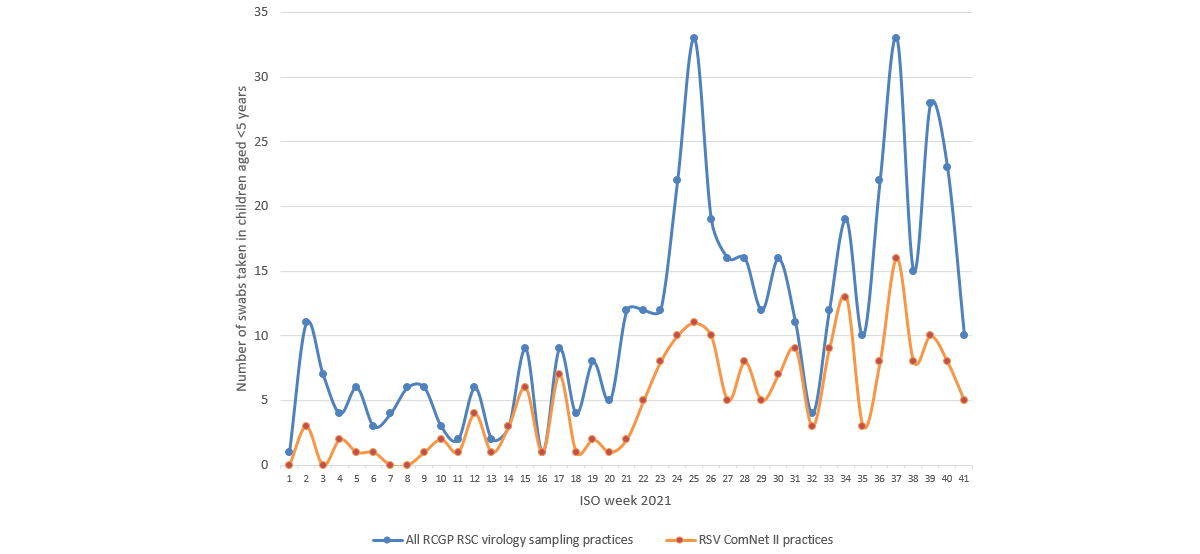
Number of swabs collected by all RCGP RSC virology sampling practices and RSV ComNet II study practices. RCGP RSC: Royal College of General Practitioners Research and Surveillance Centre; RSV: respiratory syncytial virus.

Of the 457 swabs, 200 (43.8%) were collected among children aged <5 years in the RSV study practices by the 16 practices recruited into the RSV ComNet II study thus far (see Figure 6) up to ISO week 41, 2021; of these 200 swabs, 37 were collected by RSV ComNet II study practices in the winter of 2020-2021 between ISO weeks 1-20, 2021.

The RSV swab positivity rate among children aged <5 years was 21.8% (100/457) across the entire RCGP RSC virology surveillance network, whereas an RSV swab positivity rate of 27% (54/200) was seen in the practices recruited for the study.

### Preliminary Characteristics of the Study Population From the Patient Questionnaires

Preliminary results on the demographic characteristics and clinical symptom presentation of the included study population so far are presented in Tables 2 and 3 up to ISO week 41, 2021. These results do not include data linked to the medical record. There were 45 Day 14 questionnaires in this preliminary analysis, one of which was completed using the Day 30 questionnaire and thus contained incomplete information for certain entries. There were 24 Day 30 questionnaires, one of which was completed using the Day 14 questionnaire.

**Table 2:** Demographic characteristics of patients with respiratory syncytial virus (RSV)–positive swabs.

Characteristic		Children (N=45)
Age (months), median (IQR)		20 (26)
**Age group (months), n (%)**		
	1-12	14 (31)
	13-24	12 (27)
	25-60	19 (42)
Gender, male, n (%)		22 (49)
Prematurity, n (%)		8 (18)
**Presence of chronic condition, n (%)**		
	Respiratory disease	1 (2)
	Malnutrition	Information not available for preliminary results
	Immunocompromised	0 (0)
	Others	3 (7)
Previous RSV infection in this season, n (%)		2 (4)
**RSV typing, n (%)**		
	RSV A	18 (40)
	RSV B	27 (60)
Coinfection with at least one other virus, n (%)		7 (16)

**Table 3:** Clinical symptoms of patients with respiratory syncytial virus (RSV)–positive swabs at Day 14 and Day 30.

Clinical symptoms	Day 14	Day 30	Combined
Shortness of breath, n/N^a^ (%)	18/44 (41)	1/24 (4)	N/A^b^
Cough, n/N (%)	42/44 (95)	8/24 (33)	N/A
Sore throat, n/N (%)	5/44 (11)	1/24 (4)	N/A
Coryza at Day 14 or nose complaints at Day 30, n/N (%)	20/44 (45)	8/24 (33)	N/A
Fever, n/N (%)	30/44 (68)	2/24 (8)	N/A
At least 1 persisting symptom, n/N (%)	20/45 (44)	12/24 (50)	N/A
Returned to normal daily activities, n/N (%)	42/45 (93)	20/24 (83)	N/A
Duration of illness^c^, median (IQR)	N/A	N/A	14 (13)

^a^N indicates the number of respondents from which the data are available. n indicates the number of patients with the specified symptom.

^b^N/A: not applicable.

^c^Calculated over a period of 60 days (the upper limit of Day 30 questionnaire).

## Discussion

### Principal Findings

Our results demonstrate that it is feasible to implement a standardized RSV burden of disease protocol in England during the COVID-19 pandemic. Although the pandemic has restricted access to primary health care, with more remote management of patients with respiratory symptoms and differences in the epidemiology of respiratory infections, the RCGP RSC sentinel network has acted as an adaptive platform and implemented the ComNet standardized protocol.

Of the 457 swabs among children aged <5 years, 200 (43.8%) were collected by the 16 practices participating in the RSV ComNet study up to ISO week 41, 2021; 100 were collected between ISO weeks 1-20, compared to the 382 collected in children aged <5 years between ISO weeks 1-25 in the last winter season 2019-20 and the 116 collected in children aged <5 years between ISO weeks 1-20 in the prepandemic year, 2018-19. Thus, the swabbing rate in children aged <5 year across the whole network was approximately 26% the equivalent rates for 2019-20 and 86% of equivalent rates for 2018-19.

As of ISO week 41, 2021, we were able to collect 54% (54/100) of the RSV-positive samples from children aged <5 years from an existing sentinel surveillance network in England for the RSV ComNet II study.

We noticed early in our implementation that the RSV swab positivity rate was concentrated in a small number of virology-swabbing practices across the sentinel surveillance network. This finding is similar to those in New York that show patchy RSV incidence during the COVID-19 pandemic [33]. This result has meant that recruiting practices for the study has focused on actively targeting practices that have seen recent RSV cases, which may not be generalizable to other years when the burden of RSV is more evenly spread across the network.

### Strengths and Weaknesses of Our Study

A strength of this study is that it was nested in a large sentinel network that has been undertaking primary care surveillance of respiratory illness for over 50 years [22,23].

The scientific methodology developed for this study uses integrated medical record systems to obtain virological swabbing codes that allow researchers to carry forth a comprehensive analysis. Practices within the network are regularly involved in clinical and epidemiological research, and there are high levels of research engagement across the network. Thus, practices within the network are familiar with undertaking research surveys and able to explain clinical contexts to potential research participants, which allows the study to gather high-quality virological samples that reduce false positive and negative rates commonly observed in many parts of the world.

Undertaking the study during the COVID-19 pandemic has raised the awareness of cocirculating viruses and has encouraged many practices to participate in the virology-swabbing scheme, which was facilitated by our inclusion of verbal study consent and home swabbing. Evidence around the use of home swabbing for research studies is still limited compared to in-practice swabbing [28-31]; thus, it is important to observe if there are any biases introduced as a result of these adaptions of the study. However, recent clinical guidance [5] in the United Kingdom suggests that children aged <5 years with LRTI should be seen in person, thus encouraging the swabbing of more cases in the target age group for this study.

However, undertaking the study during the COVID-19 pandemic and COVID-19 vaccine rollout has meant that practices were under an increased workload pressure, with some practices having to pull out due to a lack of capacity. The implementation of NPIs during the COVID-19 pandemic has also changed the epidemiological pattern of RSV, and thus, our results on the clinical and socioeconomic burden of RSV may not be generalizable to other years. Restrictions on access to primary care and differences in managing patients with respiratory symptoms may have an influence on health care use for patients with RSV during the pandemic. For example, more patients may seek testing for COVID-19 only, which largely takes place outside of primary care currently and may also influence the estimates of the clinical and socioeconomic burden of RSV from this study.

RSV did not follow the usual winter pattern [34,35], and the incidence rates and uneven spread of RSV cases throughout the network may be the result of differential swabbing practices due to differences in symptom severity or community prevalence of COVID-19; thus, the results may not be generalizable to other years.

Between ISO weeks 48-52, in December 2020, when RSV cases would normally have peaked [36-38], no cases of RSV were reported through national virology surveillance, with a similar lack of cases being reported by other European countries [27].

### Comparisons With Prior Work, Unanswered Questions, and Need for Further Work

It was predicted that as NPIs and travel restrictions ease, the levels of circulating RSV would increase. It has also been hypothesized that changes in health-seeking behavior during the COVID-19 pandemic could have contributed to a reduced detection of RSV and that a return to normal health-seeking behaviors would see a subsequent rise in the detection of RSV [39,40]. Furthermore, it was suggested that RSV infections could present more severely as older children, who were not initially exposed to RSV during the start of the COVID-19 pandemic, would be at increased risk of contracting a severe RSV infection [27]. Indeed, since June 2021, there has been a noted, consistent increase in RSV-positive swabs from national virology swabbing.

Future studies could provide sufficient data for a comprehensive socioeconomic analysis in relation to disease burden among children aged <5 years across European countries, which would further attest to developing cost-effective models for future RSV interventions.

### Protocol Amendments

Important protocol amendments will be referred to the English National Research Ethics Committees for ethical approval. Once approved, it will be communicated directly with the recruiting study practices. The amended protocol will be shared with all relevant parties, such as investigators and clinical research networks, in a timely manner.

### Conclusions

This study aimed to demonstrate the possibility of implementing a standardized protocol to assess the clinical and socioeconomic impact of RSV within England. Although the results may not be easily generalizable to other years due to the COVID-19 pandemic, the lessons learned and adaptations made in light of this study may still serve to inform other studies recruiting patients via the national surveillance network in England.

## Appendix

Multimedia Appendix 1 Day 14 questionnaire.

## Abbreviations

ARI: acute respiratory illness
CMR: computerized medical records
GP: general practitioner
ILI: influenza-like illness
ISO: International Organization for Standardization
LRTI: lower respiratory tract infections
Nivel: Netherlands Institute for Health Services Research
NPI: nonpharmaceutical intervention
RCGP: Royal College of General Practitioners
RSC: Research and Surveillance Centre
RSV: respiratory syncytial virus
SCTID: Systematized Nomenclature of Medicine Clinical Terms identifier

## Data Availability

Data cannot be shared publicly because of it is owned by the Oxford-Royal College of General Practitioners (RCGP) Research and Surveillance Centre (RSC) and its participating practices. Data are available from the University of Oxford Institutional Data Access Committee (contact via ku.ca.xo.chp@nanigsuled.nomis) for researchers who meet the criteria for access to confidential data. The data underlying the results presented in the study are available from https://orchid.phc.ox.ac.uk/. Further enquiries about the RCGP RSC network and data requests can be found at https://orchid.phc.ox.ac.uk/index.php/orchid-data/ or by emailing ku.ca.xo.chp@seiriuqneecitcarp.
