# Supplementary material for: Assessing the Clinical and Socioeconomic Burden of Respiratory Syncytial Virus in Children Aged Under 5 Years in Primary Care: Protocol for a Prospective Cohort Study in England and Report on the Adaptations of the Study to the COVID-19 Pandemic
Source: JMIR Res Protoc. 2022 Aug 25;11(8):e38026. doi: 10.2196/38026 (PMC9415952; doi:10.2196/38026)
Supplement: Multimedia Appendix 1 [file resprot_v11i8e38026_app1.docx]

**Multimedia Appendix 1.**

**RSV ComNet II Questionnaire (Day 14)**

**Data Linkage**

1. Practice name:
2. Patient study number:
3. Date swab was taken: (dd/mm/yyyy)
4. Please confirm study consent: (Yes / No)

**Patient Demographics (of the child)**

1. Date of birth: (dd/mm/yyyy)
2. Sex: (Male / Female)

**Date of Onset of Clinical Symptoms and Presenting Clinical Symptoms**

1. Symptom onset date: (dd/mm/yyyy)
2. Presenting clinical symptoms when swab was taken? (Please select all that apply): (Shortness of breath / Wheezing / Cough with slime / Cough without slime / Sore throat / Coryza / Fever ≥ 38 ° / Feeding difficulties / None of the above)

**Medical History of Child**

1. Birth weight (grams)?
2. Further medical history of child. (Please select all that apply): (Premature birth / Chronic respiratory disease / Immuno-comprised / Other chronic medical condition / Previous RSV infection this season / None of the above)
   1. You selected 'Premature birth', please specify number of weeks:
   2. You selected 'Chronic respiratory disease', please specify:
   3. You selected 'Other chronic medical condition', please specify:
3. Influenza vaccination this season? (September 2020 onwards): (Yes / No / I don’t know)
4. Did your child receive preventive medication this season for influenza? (E.g. Palivizumab) (Yes / No / I don't know)

**Health Care Use Related to RSV in the Past 14 Days**

Contacts with the GP/paediatrician since your child was swabbed:

1. Number of phone or e-mail contacts?
2. Number of visits to the GP/paediatrician?
3. Number of home visits by the GP/paediatrician?
4. Did your child visit another doctor since he/she was swabbed? (Yes / No/ I don’t know)
   1. If 'Yes', type of doctor? (Medical specialist / Other)
      1. If 'Other', please specify
   2. Number of visits (with Medical specialist/Other doctor):
   3. Number of home visits (with Medical specialist/Other doctor):
   4. Number of phone or e-mail contacts (with Medical specialist/Other doctor):
5. Did your child visit A&E related to the RSV infection, since he/she was swabbed? (Yes / No / I don’t know)
   1. If 'Yes', how many times did your child visit A&E?
6. Was your child hospitalized due to the RSV infection, since he/she was swabbed? (Yes / No / I don’t know)
   1. If 'Yes', number of days hospitalized?
   2. Was your child admitted to the intensive care unit (ICU)? (Yes / No / I don’t know)
      1. If 'Yes', number of days in ICU?
7. Did your child require any paramedical help related to the RSV infection, since he/she was swabbed? (Yes / No / I don’t know)
   1. If 'Yes', please specify type of paramedical help? (Please select all that apply): (Nurse / Nutrition / Physiotherapy / Other / None of the above)
      1. You selected 'Other', please specify:
8. Did your child receive any medical treatment related to the RSV infection, since he/she was swabbed? (Yes / No / I don’t know)
   1. If 'Yes', please specify type of medical treatment? (Please select all that apply): (Paracetamol / Other pain medication / Antibiotics / Nebulizers / Nose spray / Cough syrup / Other / None of the above)
      1. You selected 'Other', please specify:
   2. If 'Yes', please specify how many days was the medication used?

**Days of Illness**

1. How many days do you consider your child was ill?

**Socio Economic Impact**

1. Was you child out of day-care or school? (Yes / No / N/A (Child doesn't go to day-care or school))
   1. If 'Yes', please specify number of days out of day-care or school?
2. Which of the following situations fits your situation? (If there are multiple situations, please indicate the most common situation) (I have a full-time paid job / I have a part-time paid job / I take care of the household and children)
   1. If part-time, please specify how many hours you work per week?
3. Did you need to take sick leave due to your child’s illness? (Yes / No)
   1. If 'Yes', please specify number of days of sick leave?
4. Were you affected at work due to your child’s illness? (Yes / No)
   1. If 'Yes', please specify number of days you were affected at work?
   2. If 'Yes', please estimate the size of the impact during these days on a scale of 0 to 100? (0 = no impact, 100 = maximum impact)
5. Do you have a partner or is there another person that takes on a large part of the child's care? (If 'No', the survey will skip to 'Current Health Status') (Yes / No)

**Socio economic impact on your partner or other person that takes on a large part of the child's care**

1. Which of the following situations fits this person's situation? (If there are multiple situations, please indicate the most common situation) (They have a full-time paid job / They have a part-time paid job / They take care of the household and children)
   1. If part-time, please specify how many hours this person works per week?
2. Did this person need to take sick leave due to your child’s illness? (Yes / No / I don’t know)
   1. If 'Yes', please specify number of days of sick leave this person took?
3. Was this person affected at work due to your child’s illness? (Yes / No / I don’t know)
   1. If 'Yes', please estimate the size of the impact on this person during these days on a scale of 0 to 100? (0 = no impact, 100 = maximum impact)

**Current Health Status**

1. Has your child returned to normal activities? (E.g. day-care, pre-school etc) (Yes / No / I don’t know)
   1. If 'Yes', since when has your child returned to normal activities? (dd/mm/yyyy)
2. Has your child still got any symptoms related to the RSV infection? (Please select all that apply): (Wheezing or whistling in the chest / Persistent cough with slime / Persistent cough without slime / Nose complaints, e.g. runny nose, stuffy nose / Sore throat / Shortness of breath / Fever ≥ 38 ° / Feeding difficulties / None of the above)

**Quality of Life**

1. On a scale of 0 to 100: How good or bad your child’s health is TODAY? (0 = the worst health you can imagine, 100 = the best you can imagine)

**Complications Related to the RSV Infection**

1. Has a physician diagnosed your child with an Acute Otitis Media (middle ear) infection since your child was swabbed? (Yes / No / I don’t know)
2. Has a physician diagnosed your child with a pneumonia since your child was swabbed? (Yes / No / I don’t know)
